# What promotes sustainability in Safe Community programmes?

**DOI:** 10.1186/1472-6963-9-4

**Published:** 2009-01-08

**Authors:** Cecilia Nordqvist, Toomas Timpka, Kent Lindqvist

**Affiliations:** 1 Linköping University, Department of Medicine and Health Sciences, SE-581 83 Linköping, Sweden

## Abstract

**Background:**

The theory and practice of safety promotion has traditionally focused on the safety of individuals. This study also includes systems, environments, and organizations. Safety promotion programmes are designed to support community health initiatives taking a bottom-up approach. This is a long-term and complex process. The aim of this study was to try to empirically identify factors that promote sustainability in the structures of programmes that are managed and coordinated by the local government.

**Methods:**

Four focus group sessions with local government politicians and administrators in designated Safe Communities were conducted and analyzed using qualitative content analysis.

**Results:**

Collaboration was found to be the basis for sustainability. Networks, enabling municipalities to exchange ideas, were reported to positively influence the programmes. Personal contacts rather than organizations themselves, determine whether collaboration is sustained. Participants reported an increase in cross-disciplinary collaboration among staff categories. Administrators and politicians were reported to collaborate well, which was perceived to speed up decision-making and thus to facilitate the programme work. Support from the politicians and the county council was seen as a prerequisite. Participants reported an increased willingness to share information between units, which, in their view, supports sustainability. A structure in which all local authorities' offices were located in close proximity to one another was considered to support collaboration. Appointing a public health coordinator responsible for the programme was seen as a way to strengthen the relational resources of the programme.

**Conclusion:**

With a public health coordinator, the 'external' negotiating power was concentrated in one person. Also, the 'internal' programme strength increased when the coordination was based on a bureaucratic function rather than on one individual. Increased relational resources facilitated the transfer of information. A regular flow of information to policy-makers, residents, and staff was needed in order to integrate safety programmes into routines. Adopting a bottom-up approach requires that informal and ad hoc activities in information management be replaced by formalized, organizationally sanctioned routines. In contrast to injury prevention, which focuses on technical solutions, safety promotion tries to influence attitudes. Collaboration with the media was an area that could be improved.

## Background

Injuries are globally recognized as an important public health problem. Every year, intentional and unintentional injuries account for about five million deaths or 9% of all mortalities, a rate equal to the deaths from HIV, malaria and tuberculosis combined.[1] Also, non-fatal injuries cause considerable suffering and costs to individuals, their families and society. Consequently, there is a need for knowledge about programmes that can reduce injury rates and improve public safety. Although the terms 'injury prevention' and 'safety promotion' are often used interchangeably, [2] there are important theoretical and practical differences between the concepts. 'Injury prevention' denotes a scientifically informed 'engineering' process involving scientists and experts; prevention of motor vehicle injuries is its model application area. 'Safety promotion' is a bottom-up process of societal development which has a democratic foundation with strong lay involvement.[3]

Comparing these approaches to managing the problem of injuries, it becomes evident that the last few decades have witnessed a rapid development of the scientific foundation for injury prevention.[4] For instance, in the 1960s and 1970s, Haddon and co-workers developed a matrix for injury event analysis, in which human, vehicle, equipment, physical environment, and socio-economic factors are divided over the injury time sequence into pre-event, event, and post-event categories. This approach and conceptual framework made it much easier to analyse mechanisms behind injury occurrence and to formulate prevention programmes focused on items in the causal chains leading to injury.[5] In parallel, methods from the behavioural sciences were applied to analysis of injury events.[6] Programmes that consider both individual behaviour and the social and physical environments have been developed since the 1980s [7], and ecological study design, which is consistent with a public health approach, is increasingly applied in injury research.[8]

The theoretical and practical aspects of safety promotion have not been advanced to the same level. However, in developing health policies it is increasingly recognized that health and safety programmes must be planned and operated in a societal context, while not excluding the level of the individual.[9] Both the Ottawa Charter and the Sundsvall Statement have highlighted the association between the social and cultural environments, health and safety.[9] Theoretical and practical interest is thereby extended beyond the individual and personal risk factors to include the system, environment and organization.[9] One way to summarize this shift is to say that safety promotion programmes are designed to support 'community development for health', [10] that is, to support entire communities as they address their specific local injury problems by developing appropriate structures and mobilizing local resources from the bottom up.

### Safe Communities

Reports indicate that injuries among children and working-age adults and injuries related to violence are decreasing in countries with increasing gross national product (GNP), while injuries due to falls among the elderly and to suicide acts are increasing.[11] The overall health status in a country has also been associated more with the distribution of wealth in the population than with the absolute GNP.[12] Consequently, injury rates do not have a simple connection to economic development, and injury control therefore poses different problems in different countries. The Safe Community movement was developed in the 1980s, based on the idea that local communities can identify their injury problems and take action accordingly. The first Safe Community, Lidköping, Sweden, was designated in 1989. There are at present (i.e., in June 2008) more than 130 designated Safe Communities in the world and their number is increasing. These communities have signed a contract with the World Health Organization (WHO) Collaborative Centre on Community Safety Promotion at the Karolinska Institute in Sweden, agreeing to work according to six indicators for Safe Communities. The indicators are: [13].

1. An infrastructure based on partnership and collaborations, governed by a cross-sectional group that is responsible for safety promotion in their community;

2. Long-term, sustainable programmes covering both genders and all ages, environments, and situations;

3. Programmes that target high-risk groups and environments, and programmes that promote safety for vulnerable groups;

4. Programmes that document the frequency and causes of injuries;

5. Evaluation measures to assess their programmes, processes and the effects of change;

6. Ongoing participation in national and international Safe Communities networks.

The second indicator: "Long-term, sustainable programmes covering both genders, all ages, environments and situations" is the focus of the present study.

### Contribution of the programmes

Research performed during the last few decades has shown that preventive measures can reduce the impact of injuries on communities.[4,14] In Sweden the mortality from injuries decreased by one-third between 1972 and 2002. This reduction has been attributed mainly to successful traffic injury prevention programmes.[15] It is less evident, however, how much safety promotion programmes contribute to the reduction in mortality and morbidity. Looking at the degree of concentration on community development and local injury problems, we see that designated Safe Communities do not necessarily have lower total injury rates than non-designated municipalities.[16] One explanation for this finding is that municipalities starting to work in programmes to become designated Safe Communities had higher injury rates to begin with. Another explanation may be that the right questions are not being asked when evaluating collaborative activities.[17] It is also possible that interventions that are effective in subgroups may not reduce the total injury rates in a municipality. A final possible explanation, which is perhaps the most important, is that the community development process is complex and time-consuming.[18] It can take 10 to 15 years to implement a community-based programme.[19] Complicating matters is that the authorities seeking to make interventions have themselves contributed to shaping the municipality's current health situation.[20]

Member countries of the WHO have accepted responsibility for promoting and protecting the health of their populations by ensuring that sustainable health systems are accessible to all.[21] Our hypothesis is that sustainable structures which allow implementation of safety promotion interventions are a prerequisite for improved security and a higher sense of safety for citizens. Research on factors that support sustainability in the programmes is as yet limited.[22] Only one previous relevant study was found. The study concluded that interrelated factors, such as financial resources, human resources, and relational resources including inter-sectoral collaboration, are important for sustainability. By contrast, injury surveillance and goal formulation appeared to have less influence on sustainability. Feedback on how to improve the programmes and maintain long-term effectiveness was found to be minimal.[23] Consequently, the present study has focused on empirically identifying factors that promote sustainability in the structures of programmes that are managed and coordinated by the local government.[20]

In efforts to reduce injury rates there is a difference between bottom-up and top-down approaches to analyses and development. There has been little clarification on how to make the Ottawa Charter's concept.[24] of empowerment operational and effective in the programmes aimed at reducing injury rates.[3] The aim of this study was to examine factors that are perceived by local politicians and local government officials to make safety programmes sustainable. In the study, safety promotion programmes are represented by designated Safe Communities in Sweden, where the programmes are coordinated by local governments, mainly in municipal administrative offices.

## Methods

"Long-term" is not defined in the description of the indicators, thus we will examine subjective perceptions of long-term, or sustainable, safety promotion programmes. We chose to collect our data using focus group sessions so that we could access different qualities of safety promotion knowledge accumulated by the participants – officials and politicians with experience of safety promotion work in Safe Communities.

### Study population

To gather data from subjects with extensive experience of safety promotion work, we invited the first ten municipalities designated as Safe Communities in Sweden to participate in the study. All of the invited municipalities agreed to participate. Five municipalities designated between 1989 and 1997 were allocated to one group, and five municipalities designated between 1995 and 2000 to another group. There was an overlap of years because we wanted the focus groups to be as similar as possible concerning the number of inhabitants and geographical location. The safety promotion work had been initiated about 10 years before the designation. The first group will be referred to as 'ed municipalities' (ed = early designated), and the second as 'ld municipalities' (ld = later designated). The ed group contained four medium-sized and one sparsely populated municipality, while the ld group contained one suburban, one sparsely populated, one industrial, and two large municipalities. Four of the ed municipalities had a decreasing in-patient injury trend, while three of the ld municipalities had an increasing number of in-patients between the years 1987–1989 and 2000–2002.[25] Different parts of Sweden were represented, but with a focus on the country's south-west. Injuries were defined as physical, non-chronic, unintentional or intentional injury or injury due to unclear intention, according to the International Classification of Diseases, 10th revision (ICD-10).

### The Swedish context

In Sweden, municipalities and county councils rule by self-government, and politicians are elected in general elections. Local authorities are responsible for shaping the welfare system in their particular municipality or city council, according to local conditions. Most income tax revenues are used for activities run by local authorities. The municipalities are responsible for implementing a wide range of welfare state services, from care of the elderly and disabled to support for the rehabilitation of injured persons and post-medical care. Further, local government activities are restricted by national legislation.[26] For instance, all municipalities are obliged to have a programme for the coordination of the Civil Protection Act against Accidents. The goal of this 2004 Act is to protect human life and health from accidents and to minimize damage to property and the environment.[27]

A municipality's decision to try to become a designated Safe Community is made at the political level. In the beginning of the Safe Community process, it is thus essential to gain political support whether or not the initiative to become a Safe Community originates among the local politicians or local officials.

Every local administration in the municipality, under its political board, is responsible for financing safety work. When a Safe Community is initiated, a special Safe Community budget is usually created. The budget may fund a public health coordinator, for instance. In many older Safe Communities, the safety work has been integrated into other responsibilities of the administration.

### Data collection

The research team planned four focus group sessions with five participants in each group, one with local government officials from ed municipalities, one with local government politicians from the same communities, one with officials from ld municipalities, and one with local government politicians from the same municipalities.

The interview guide contained questions about how the participants defined success in community-based safety work, what was perceived to work and not to work in the municipality, perceived support for safety work, networks, the impact of contextual factors, and evaluations.

In March 2007, one of the authors moderated the four sessions. An observer helped, prompting with questions that had not been covered, at the end of the session, according to an established procedure.[28,29] often used by the moderator. All officials participated in focus group sessions and three politicians participated in each politician focus group session. The semi-structured sessions were audio-recorded. Shortly after the focus group sessions, the moderator conducted telephone interviews with the remaining four politicians using the same interview guide as in the focus groups and giving the participants as much time as they wanted to formulate their answers.

### Analysis

We conducted a qualitative content analysis [30,31] in order to describe and understand the data. All sessions were transcribed verbatim. The first author (C.N.) read the texts through several times. She then organized the texts in order to abstract sustainability in relation to municipality safety promotion. For the present paper, meaning units [32] (selected parts of the texts that are relevant for the purpose of the study) dealing with sustainability in the safety promotion programmes were selectively coded. Each code described the meaning unit in its context. For instance the code "continuous back-up" describes the meaning unit "There are constantly new parents, but the information must live on. It is important for staff that they get back-up, input and pushing so that they do not think that; no this we have kept on repeating for five and ten years. There are new people in front of us so it is still important that the message becomes part of the consciousness." Both manifest statements about sustainability and the author's latent interpretations of factors that could affect sustainability were selected.[30] The codes were then used to facilitate grouping the meaning units, with their contexts, together into the following eight categories: collaboration, municipality administration, funding, attitude, public health coordinator, information, visible profile and integration into routines. We tried to create distinct categories, each comprising an individual meaning unit that would not fit into another category. The categories are developed further in the results section.

The reliability was tested by intra-rater reliability. The first author tested her own results by repeating part of the coding. Inter-rater reliability was not tested, but the results were discussed among a group with extended experience of safety research consisting of researchers and representatives from the Swedish National Institute of Public Health, the national Board on Health and Welfare, the Karolinska Institute and Umeå University. All authors agreed on the findings.

Officials' and politicians' statements were kept apart at the beginning of the process, but are here presented jointly. Statements from ed and ld municipalities are presented separately where there are differences.

## Results

The ideal community size for implementation of a safety promotion programme was reported by the respondents to be a 'medium-sized Swedish municipality', that is, a community with 20,000–50,000 inhabitants. Most municipalities of this size were reported by the representatives from the ed municipalities to have a more integrated safety network compared with adjacent, smaller municipalities, and to be big enough for a sufficient number of discussion partners. Ld municipalities suggested that a medium size is ideal because it is large enough to have resources for a public health coordinator and still small enough to perform safety audits and survey public opinion.

With respect to safety promotion, the participants believed that it is important to focus on the special problems of a municipality, which may range from the traffic situation to drowning accidents. Therefore, methods may vary from one municipality to another even though the municipalities may appear to share the same safety goals.

### The local government's interaction with residents through intermediaries

Both municipality groups believed that efficient interaction between the municipal administrators and residents is important for sustainability of the programmes. They had channelled the safety work through a number of organizations in both the public and private sectors; some of these had their own funding. Consequently, there was an ongoing process by which certain organizations and professionals acted as intermediaries between the programme managers and the residents. Mentioned examples included staff involved in geriatric injury prevention, and organizations which provided the means for two-way communication with the elderly population. Other organizations mentioned were the Red Cross, churches and schools.

In the ed municipalities, the communication channels that had been established between the municipal administration and the residents were mainly described as making it possible to inform the public about the connection between behaviour and risk of injury. This was perceived to be a successful structure for distributing injury prevention information, for example, through children's health centres and schools.

### The local government's interaction with other institutional actors

The reports of collaboration with national institutions such as the Swedish National Institute of Public Health and the Swedish National Rescue Service Agency were mixed in both municipality groups, some being described as supportive and others as lacking engagement. The Karolinska Institute's role was described as unclear. Ld municipalities reported collaboration problems with the National Rail Administration and the National Road Administration. Personal contacts rather than the organization itself determine whether the collaboration works and is sustained, according to the participants from ld municipalities. Networks and visits between municipalities were reported to positively influence the safety work by facilitating the exchange of ideas.

A general feeling of security among residents was emphasized by both municipality groups as perhaps the most important aim of safety promotion. In other words, crime prevention was an important part of the programmes and the police were seen as central collaborative partners. The need for local police offices was discussed in the ld municipalities. In urban planning, crime prevention was considered, as reported by the ld municipalities. The county councils and the municipalities were perceived to have common interests and therefore cooperation between these institutions of local government was seen as important for sustainability. According to ld municipalities, cooperation had been better in the past.

#### Funding

Focused investments in key areas, especially at the beginning of the process, were considered essential for the establishment of safety programmes. One example mentioned was the area of traffic safety; a structure was in place for traffic safety activities before the designation, but this area still received special funding. Therefore, the traffic safety activities were considered external to the Safe Community programme. The participants from ed municipalities reported that they had received financial support from the county council, which was perceived to be important since the municipalities often lacked their own funding for safety work. It was therefore perceived to be important for politicians and officials to clearly demonstrate the political will to support safety promotion at the county level in order to encourage funding.

### Organization of safety promotion at the municipality's administrative office

#### Budget

Both municipality groups believed that the most sustainable model was one in which the councils for each local safety area were responsible for funding their own safety promotion work. The participants recognized the local economic advantages of preventing injuries, and discussed how funding was dependent on evaluation processes. However, these evaluation processes were dependent on injury registration, something that was reported not to work well in all municipalities. Thus, there was a vicious circle, according to the participants; with no evidence of benefits, funding was withdrawn, while procuring evidence was an activity that required funding.

#### Collaboration between local government administrators and professional staff

Cross-disciplinary collaboration within the municipal administration was perceived to be essential for sustainable safety programmes. An increase in collaboration between staff categories had been observed in the municipalities, both among administrators and between professionals belonging to other staff categories, for example, the home help service and the police. A structure in which all local authorities' offices are in close proximity was seen as supporting collaboration and administration of community work, including safety promotion in the ld municipalities. Both municipality groups perceived that efficiency increased when different local government committees worked together instead of defending their own territories, and when they shared a safety promotion perspective. Both groups reported a positive trend toward changing responsibility from project groups, depending on the enthusiasm and commitment of employees, to being integrated into the municipality's routines. This ensured that the project was on solid ground for continuation even if individual officials left their positions. General commitment and enthusiasm in the different local government sectors and in health care were still emphasized as prerequisites for sustainability.

Participants in the ld municipalities asked for a functioning organizational model into which safety promotion could be integrated. One aspect of integration discussed by the ed municipalities was that already integrated safety promotion implementations can turn invisible when employees start taking them for granted, and act without reflecting on safety. This can be a dilemma. On the other hand, according to the participants, selective measures were not the goal; rather, they aimed for a continuing integrated safety promotion process.

#### Political commitment and collaboration with municipal officials

A positive attitude and good top-down support from administration managers and politicians is essential for carrying through bottom-up propositions, according to both municipality groups. Though the officials were responsible for the safety promotion, overall support from the politicians and the county council was viewed by the participants from ed municipalities as a prerequisite for satisfactory functioning. Officials and politicians were reported by both municipality groups to collaborate well, in an open atmosphere, which was perceived to speed up decision-making and thereby facilitate the safety work. Also, different political parties were reported to agree on safety promotion work. It was perceived as important in the ed municipalities that politicians participate in meetings at all administrative levels to show that they place a high priority on safety promotion.

The participants observed that administration directors were becoming more positive toward safety promotion after the cutbacks in the 1990s. Participants from the ld municipalities identified a risk of a politician's interest waning as soon as other issues become more pressing.

#### A public health coordinator

The safety promotion concept is widening, according to the participants from ld municipalities: from issues concerning the elderly, children and traffic safety it has broadened to include crime prevention, sports safety and sometimes mental health. Therefore, the number of collaboration partners is increasing, according to the participants. Both municipality groups identified the introduction of a public health coordinator as one means to address the growing needs and to guarantee sustainability. In some cases this is a full-time position and in other cases it would be part-time. This position would be funded by the local authorities, the county council or both.

#### Information management and marketing strategies

The participants believed that a systematic routine for management of information is essential for programmes to be sustainable. The professional staff needed efficient means for distribution of information. They needed to be able to regularly notify key groups of citizens, for example, the elderly or parents, about injury hazards. It was also thought necessary to support the politicians with updated information in order to keep up their interest and confidence in the concept of the Safe Community. To maintain sustainability, politicians need to be able to adapt safety promotion interventions to events and trends in society. Participants reported a general trend in units being more willing to share information, including information in the field of safety promotion. This willingness was perceived to support sustainability of the safety promotion programmes.

Strategic management of the Safe Community image was perceived as important. The image of the community as a safe place to live was reported to be of value, for example, when foreign companies wanted to establish themselves in the municipality. It was felt that the local media could support distribution of information to the public; however, both community groups reported that cooperation with the media was not optimal at present. They felt that the media could give greater support by reporting more of the daily safety work. Participants also indicated that sustainability would be supported by giving the municipality's status as a Safe Community a high profile, ensuring that residents are aware of it.

### The future

According to both Safe Community groups, designating a community a Safe Community for a new period can be discussed from two perspectives. On the one hand, the process is time-consuming, and a sustainable local organization for safety promotion is more important than the publicity that a renewed designation may bring. On the other hand, if the community is not re-designated a Safe Community, the municipality is excluded from the Safe Community network. Also, the process in itself is beneficial in the sense that it clarifies to those involved what has been achieved, and how.

An increasingly positive image of public health work in society was reported in the sessions. This should be considered a support for sustainable programmes in all spheres.

### Differences between the municipality groups

Some matters were discussed in only one of the municipality groups. In the ed municipalities, communication between the municipal administration and the residents was described as a means of informing the public. These municipalities also discussed the importance of support from politicians and the county council for a proper functioning of the safety promotion work. In the ld municipalities, collaboration between the local and national administration was considered to depend on personal contacts. Compared with the ed municipalities, there was greater emphasis in these municipalities on local police and crime prevention. They also emphasized geographical proximity of different offices as important for collaboration. The ld municipalities discussed the risk of implementations of safety promotion measures becoming invisible when they are integrated into the daily routines. They also noticed that the safety promotion concept is widening.

## Discussion

The aim of this study was to identify factors that maintain sustainability of safety promotion programmes coordinated by municipal offices. We found that a chain of factors associated with collaboration was the basis for long-term success of these programmes. The decision-making process in the programmes was found to be horizontal, that is, the participating organizations had their own agendas and interpretation of policy, and so the municipal officials had to negotiate with the other organizations when deciding on and implementing interventions.[21] The appointment of a public health coordinator within the municipal administration, who is charged with responsibility for the programme, was seen as an effective way to strengthen the relational resources of the programme. The 'external' negotiating power of the local government was then concentrated in one person who could negotiate settlements and keep relations with other local organizers in a way that is optimal from the perspective of the safety promotion programme. The strength of the 'internal' programme increased when the coordination was based on a bureaucratic function in the municipal administration, rather than on one individual.[23] In addition, if the function is institutionalized, changes in personnel, whether municipal officials or politicians, have less impact on the programme.

Parsons' functionalist theory suggests that we have to consider the motives and values underpinning behaviour, both at an individual and collective level, when analysing social structures and processes.[33] To institutionalize a social structure for safety promotion, enough people must be motivated to comply with its requirements; also, the culture must permit people to take the necessary actions.[33] Commitment and agreement among local politicians and a positive attitude among other local policy-makers are seen as prerequisites for establishing essential relational resources during the initiation of health and safety promotion programmes.[21] Our results suggest that this commitment is just as important once the programme is running. Our study found that physical proximity between the offices of the local injury councils, the local government officials and politicians was particularly beneficial for the relational resources. The reinforcement of the relational resources around safety issues in a community can have two positive implications: more rapid and effective decision-making processes, and expansion of the network of organizations and individuals interacting to prevent injuries. The latter general expansion of the 'relational space' had increased the inter-sector trust, and seemed to have stimulated the individual actors to act toward shared goals in the present study.

One immediate result of an increase in relational resources is the facilitated transfer of information. A regular flow of information to policy-makers and staff about the programme and local injury hazards is a prerequisite for integration of safety programmes into the municipality's routines. Distribution of programme management information, at the right level of detail, has been reported to be difficult in the Safe Communities in Sweden.[34] The same study also reported a need for a group communication system that could be used to create inter-organizational trust among the organizations engaged in safety promotion. Politicians need continuous information in order to promote a programme. Only then can they effectively encourage professional staff in their safety promotion efforts by participating in meetings at all administrative levels. This is a critical measure that can improve sustainability of the programmes. Therefore, information management is an area where informal and ad hoc activities should be replaced with formalized, organizationally sanctioned routines. A risk seen in the results could be avoided by introducing functioning information routines; too effectively implemented safe promotion interventions may make the programme invisible and therefore difficult to evaluate. Evaluations were not satisfactorily performed in most of the participating municipalities, which is a well-known problem.[4,34]

In the ed municipalities, the interaction with residents was seen not only as a way to inform the politicians about the prevalent opinions in the community, but also as a possibility to inform the community about ongoing injury prevention activities. The latter may be seen as demonstrating a top-down approach to safety management. To achieve the desired bottom-up perspective, encouraging community involvement, it is necessary to actively create and maintain routines for balanced and effective two-way communication with the residents. Residents who are proud of their municipality may feel even more content and safe when they learn that safety is being promoted. Awareness of this self-enforcing loop was emphasized by both politicians and administrators.

In contrast to injury prevention, which focuses on technical solutions, safety promotion tries to influence attitudes. In this context, the media have a potential to support the programmes. In light of the importance attached to the marketing of the programme, our results suggest that collaboration with the media is an area that can be improved. The potential of the media to bring safety issues to the public's attention and to influence opinion has been recognized.[21] Also, the media may be used by public health pressure groups to influence decision-makers.[21] In Sweden the media are normally considered trustworthy, and the lack of media support that was reported here may have been due to a lack of strategies to keep the media interested. Generally, information that markets the safe community profile may help support sustainability.

Cross-sectoral collaboration and trust also facilitates funding of interventions. An overview is essential since costs incurred in one sector are often managed in another.[19] In the present study, trust through personal contacts seemed to be the only way to interact fruitfully with national institutions. Nevertheless, despite the bottom-up approach of implementing the programme, we found that the availability of specific funds still strongly influenced the safety promotion activities. Certain injury prevention areas, such as falls among elderly individuals, received funding from national agencies, which therefore indirectly determined the target of the safety work. The politicians also tried to identify and adapt to events and trends in society, which is necessary for new funding of important areas. This is a driving force which probably facilitates sustainability of the programmes, since they have to constantly adapt to the present reality. For legislative and financial resources, a top-down structure with formal actors (national and local authorities and the administration) is needed, but they can cooperate with informal actors (voluntary organizations and individuals) that take a bottom-up approach.[3] Therefore, different infrastructures derived from different institutions at different levels have to be managed at the same time.[35]

### Study limitations

In the discussions, politicians and officials from the same municipality groups brought up similar but also different factors. Thus each complemented the information supplied by the other municipality group. The aim was not to account for the differences. We do not know what the participants in one municipality group would have said about a topic discussed only in the other group. Since the ed and ld municipalities mostly represent decreasing and increasing injury rates, respectively, a future study should focus on the differences in order to find factors that promote decreased injury rates.

In the focus group discussions the participants may have restricted their comments to what they thought would be acceptable to the group.[36] There was a great deal of agreement among the participants. It may be difficult to judge whether this was in fact the result of coercion or self-censoring.[37,38] Since the atmosphere in the groups was relaxed (being fairly informal) and since the participants were used to discussions and to putting across their views, we do not think that the group setting inhibited the sharing of information. On the contrary, we believe that the discussion facilitated the expression of different points of view, as experienced in other studies.[39]

During the sessions the participants were allowed to talk freely, within the topic of the study, and also to bring up new subjects. Nevertheless the themes brought up by the moderator probably influenced the discussions. Other themes in the interview guide could have focused on other sustainability factors. Further research may reveal new factors and conditions. Psychological suffering was discussed in the sessions, but the focus was on physical injuries. We did not find any differences in results between the politicians' focus group sessions and the four telephone interviews with politicians.

Discussing the effects of the programmes was not within the scope of this study. In the future, when, we hope, programmes will be better evaluated than today, we believe that the factors for positive effect and sustainability will be equal.

## Conclusion

In conclusion, it was found that sustainable safety promotion work coordinated by municipal offices can be enforced through strengthening the relational resources, both within the municipality and from external contacts, by means of

• appointing a public health coordinator;

• ensuring that the offices are located in close proximity to one another; and

• ensuring that the information is effectively disseminated, both within the municipal administration and to residents of the community.

## Authors' contributions

TT and KL participated in the design of the study. CN carried out the data collection and analysis. CN and TT drafted the manuscript. All authors, CN, TT and KL, have critically reviewed the manuscript and have given final approval of the version to be published.

## Pre-publication history

The pre-publication history for this paper can be accessed here:


